# Supplementary material for: Drug repurposing for aging research using model organisms
Source: Aging Cell. 2017 Jun 16;16(5):1006–15. doi: 10.1111/acel.12626 (PMC5595691; doi:10.1111/acel.12626)
Supplement: Supplementary file 7 — Data S1 Zip‐Archive of all report cards. [file ACEL-16-1006-s007.zip › RC_FTB.pdf]

FTB

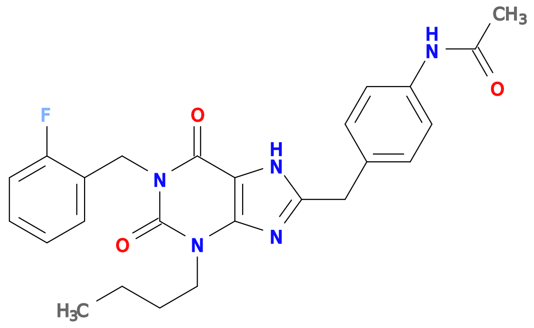

Database identifiers

ChEMBLCompound	CHEMBL120708
DrugBank	DB02008
ZINC	ZINC15804848
eMolecules	31230434
eMolecules	36366962

Ranking

	Rank	Score
Drosophila	335/697	0.455
C. elegans	169/591	0.267

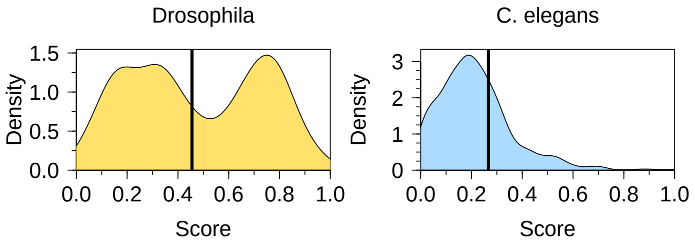

	Ageing implication	Domain conservation	Binding site conservation	Binding affinity	Bioavailability	Lipinski	Promiscuity	Purchasability	Drug approval	**Total**
Drosophila	0.36	0.951	1.0	0.912	(0.9)	0.0	-0.0	0.1	0.075	0.455
C. elegans	0.36	0.913	0.99	0.912	0.311	0.0	-0.0	0.1	0.075	0.267

Names

- 1-(2-fluorobenzyl)-3-butyl-8-(n-acetyl-4-aminobenzyl)-xanthine
- N-4-3-butyl-1-(2-fluorobenzyl)-2,6-diketo-7H-purin-8-ylmethylphenylacetamide
- N-4-3-butyl-1-(2-fluorophenyl)methyl-2,6-dioxo-7H-purin-8-ylmethylphenylacetamide
- N-4-3-butyl-1-(2-fluorophenyl)methyl-2,6-dioxo-7H-purin-8-ylmethylphenylethanamide
- N-4-1-(2-fluorobenzyl)-3-butyl-2,6-dioxo-2,3,6,7-tetrahydro-1h-purin-8-ylmethyl-phenyl-acetamide

Roles

ChEBI entry None has no roles

Status

Approved drug (according to ChEMBL)	No
Classification (according to DrugBank)	experimental
Number of Rule of 5 violations	0
Binding affinity to original target in log units (RF-Score prediction)	7.34
Burns *C. elegans* bioavailability prediction	-0.1

Compound Target Characteristics

Phosphoenolpyruvate carboxykinase, cytosolic [GTP]

Best gene implication in ageing for this target family came from gene Q9Z2V4 via mapping the annotation from Ensembl ENSMUSG00000027513 via mapping the annotation from EntrezGene 18534 via mapping the annotation from GenAgeModels 0497 annotated in GenAge release 17.

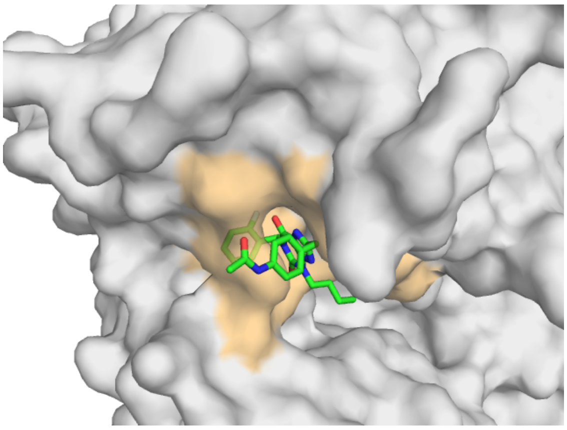

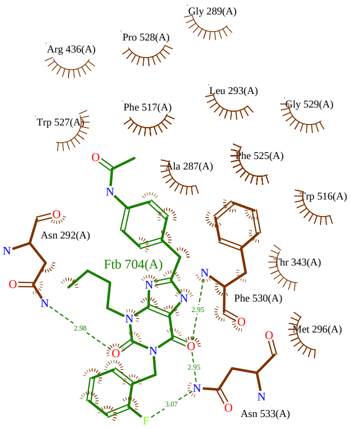

protein	amino acids contacts (binding site)
PDB:1nhx:chainA:P35558	A G N L M T R W F F W P G F N
sp:P35558:PCKGC_HUMAN	A G N L M T R W F F W P G F N
sp:P07379:PCKGC_RAT	A G N L M T R W F F W P G F N
sp:Q9Z2V4:PCKGC_MOUSE	A G N L M T R W F F W P G F N
tr:A8DYI3:A8DYI3_DROME	A G N L M T R W F F W P G F N
tr:Q7JXB5:Q7JXB5_DROME	A G N L M T R W F F W P G F N
tr:Q8ITY1:Q8ITY1_CAEEL	A G N L L T R W F F W P G F N
tr:Q8ITY2:Q8ITY2_CAEEL	A G N L L T R W F F W P G F N
tr:Q7JKI3:Q7JKI3_CAEEL	A G N L L T R W F F W P G F N
tr:Q8ITY0:Q8ITY0_CAEEL	A G N L L T R W F F W P G F N
tr:Q7JKI2:Q7JKI2_CAEEL	A G N L L T R W F F W P G F N
tr:O02286:O02286_CAEEL	A G N L L T R W F F W P G F N
tr:O44906:O44906_CAEEL	A G N L L T R W F F W P G F N

protein	whole protein ident	simil	domain-based ident	simil	contact-based ident	simil
PDB:1nhx:chainA:P35558	0.99	1.0	0.99	1.0	1.0	1.0
sp:P35558:PCKGC_HUMAN	1.0	1.0	1.0	1.0	1.0	1.0
sp:P07379:PCKGC_RAT	0.91	0.98	0.92	0.98	1.0	1.0
sp:Q9Z2V4:PCKGC_MOUSE	0.91	0.98	0.92	0.98	1.0	1.0
tr:A8DYI3:A8DYI3_DROME	0.6	0.8	0.7	0.9	1.0	1.0
tr:Q7JXB5:Q7JXB5_DROME	0.61	0.83	0.7	0.9	1.0	1.0
tr:Q8ITY1:Q8ITY1_CAEEL	0.32	0.48	0.54	0.81	0.93	0.99
tr:Q8ITY2:Q8ITY2_CAEEL	0.49	0.72	0.57	0.84	0.93	0.99
tr:Q7JKI3:Q7JKI3_CAEEL	0.55	0.81	0.59	0.87	0.93	0.99
tr:Q8ITY0:Q8ITY0_CAEEL	0.49	0.73	0.5	0.71	0.93	0.99
tr:Q7JKI2:Q7JKI2_CAEEL	0.55	0.83	0.59	0.87	0.93	0.99
tr:O02286:O02286_CAEEL	0.54	0.82	0.59	0.87	0.93	0.99
tr:O44906:O44906_CAEEL	0.53	0.8	0.57	0.84	0.93	0.99
